# Reduction of Hydrogen Cyanide in Cassava Peel With Sodium Bicarbonate and Heating Using a Stirrer Chamber Unit

**DOI:** 10.1155/tswj/6132952

**Published:** 2026-04-19

**Authors:** Narwati, Setiawan, Suryono Hadi

**Affiliations:** ^1^ Department of Environmental Health, Health Polytechnic of the Ministry of Health Surabaya, Surabaya, Indonesia

**Keywords:** cyanogenic glycosides, hydrogen cyanide, *Manihot*, temperature

## Abstract

Cassava peel, a by‐product of cassava root processing, can be used as food if processed properly. It is important to note that the high‐cyanogenic glycoside content in cassava plants has the potential to produce hydrogen cyanide (HCN), which has been proven to cause health problems. Therefore, it would be beneficial to consider a method that could help reduce HCN levels. This study explored the impact of sodium bicarbonate (NaHCO_3_) and heating on the HCN of cassava peel during the initial stage of food processing. This study is experimental in nature, as a follow‐up to previous research. The cassava tuber skin was separated from the outer layer with care, then washed thoroughly and sliced 2 mm thick. The experiment was conducted on 20 g of cassava peel, with the addition of 30 g and 60 g of NaHCO_3_, and a heating temperature of 55°C for 15 min of exposure. The results showed that the group of cassava peel samples with a dose of 60 g of NaHCO3 heated at a temperature of 55°C showed the lowest HCN content, namely 0.193 ± 0.015 mg/100 g from the initial HCN of cassava peel of 1.6024 mg/100 g. This indicates a reduction of 1.409 (87.9%), which is a significant finding. In conclusion, it appears that a combination of NaHCO_3_ and heating at 55°C could be considered as a method to reduce HCN levels in the preprocessing stage of cassava peel.

## 1. Introduction

Cassava (*Manihot esculenta*) is a staple food crop that is widely grown and used around the world, particularly in tropical regions such as South America, Africa, and Asia. All parts of the cassava plant can be utilized. As well as being used as a raw material for ethanol production, cassava peel is a source of dietary fiber and is therefore used as animal feed or processed into snacks such as cassava peel chips [1, 2]. The utilization of cassava peel can help to reduce waste and enhance the sustainability of cassava production. Cassava peel has potential economic value as a food ingredient, but it requires processing due to its high antinutrient content. Cassava roots also contain the toxic chemical compounds linamarin and lotaustralin, known as cyanogenic glycosides [3, 4]. Cyanogenic glycosides are a large group of plant secondary metabolites consisting of aglycones. Nonsweet aglycones have an *α*‐hydroxy nitrile type and a sugar group (glycone) bound through a glycosidic bond [5]. Cyanogenic glycosides found in vacuoles differ from hydrolytic enzymes (*β*‐glucosidase) found in the cytosol In the case of cellular disruption, glycosides are metabolized into sugars and cyanohydrins, which are then degraded into hydrogen cyanide (HCN) and residual aldehydes or ketones [6].

Cyanide can be formed naturally or artificially and has a strong and rapid toxic effect. It is a fact that HCN is found in relatively high concentrations in several plant species, such as cassava [7]. Nyirenda explains that every variety of cassava contains cyanogenic glycosides, although in varying concentrations [6]. Yadnya and Trisnadewi elucidated that the ratio of HCN levels in tubers and leaves is, on average, 1:2 [8]. Despite the existence of varieties of cassava with low HCN levels, the consumption of cassava can also present potential health risks. The reference limit set by FAO/WHO and SON for HCN is 1 mg HCN per 100 g of body weight. However, the cassava sample is considered safe for consumption because the concentration of HCN does not exceed the lethal dose threshold of 5–10 mg HCN/100 g of body weight [9]. Consumption of cassava containing HCN in amounts exceeding the body′s tolerance threshold can trigger various adverse health effects [10].

Pretreatment methods for cassava have been applied by previous researchers to reduce cyanide acid levels. These methods include fermentation by soaking in water ([11–13], soaking in a NaHCO_3_ solution [14], and drying and heating techniques [15]. Anjani′s study showed a positive correlation between heating time and cyanide reduction, with a statistically significant decrease of 3.38 ppm. The use of NaHCO_3_ solution at a temperature of 30–60°C resulted in faster and greater water absorption compared with NaCl of the same concentration [16]. This study indicates that there is a change in cell membrane permeability and an increase in gas pressure inside the cell, which has the potential to cause cell hydration, leading to accelerated evaporation of HCN in the cell. Therefore, a combination of chemical and physical methods, namely the use of NaHCO_3_ and heating, is considered more effective in reducing cyanide levels in cassava peel. This study is aimed at testing the effect of the mass of NaHCO_3_ solution accompanied by heating at a temperature of 55°C on the HCN content in cassava peel.

## 2. Material and Methods

### 2.1. Sampling

Samples were purposively collected from a bitter cassava (*Manihot esculenta*) variety locally known as Genderuwo. Only samples with an initial HCN concentration greater than 1 mg/100 g were included in the study. The baseline HCN concentration of the selected samples was 1.6024 mg/100 g.

### 2.2. Collection of Cassava Peel

Cassava peel samples were collected from a bitter cassava species cultivated in Wonosari Regency, East Java, Indonesia (8.097155° S, 112.534534° E). Sampling was conducted at 06:00 a.m. to maintain sample freshness and integrity. Fresh cassava tubers were harvested directly from the plantation.

The cassava tubers used in this study had an average stem circumference of approximately 30 cm, measured using a portable measuring tape. Tubers were harvested by carefully excavating the surrounding soil to minimize damage to the root system. The soil was initially loosened using a hoe, after which the tubers were manually extracted and separated at the base near the stem.

The harvested tubers were placed in plastic containers, stored in sample boxes, and transported to the Environmental Health Laboratory, Surabaya, Indonesia, for pretreatment and HCN analysis.

### 2.3. Sample Processing

A total of 25 samples were analyzed in this study. The selection of NaHCO3 concentrations (30 and 60 g per 500 mL) was based on previous findings, which reported that a 20% NaHCO3 solution reduced HCN levels by 84.22% after 12 h of immersion [17].

Samples were grouped according to NaHCO3 mass (0, 30, and 60 g) and treatment conditions, applied before and after heating at 55°C. Each treatment was performed in five replicates. Prior to treatment, cassava tubers were washed thoroughly under running water. The outer skin was removed, and only the inner portion of the cassava peel was used. The peel was sliced into uniform pieces (≤ 2 mm thickness and approximately 3 cm length).

For each treatment group, 50 g of cassava peel was weighed. The control group (KM_0_T_0_) consisted of 50 g of cassava peel immersed in 500 mL of distilled water and stirred at room temperature for 15 min at 150 rpm. For treatment group M_1_T_0_, 50 g of cassava peel was immersed in a solution containing 30 g of NaHCO3 in 500 mL of water and stirred for 15 min at room temperature. Treatment group M_2_T_0_ followed the same procedure using 60 g of NaHCO3. For heated treatments, group M_1_T_1_ consisted of 50 g of cassava peel immersed in 30 g NaHCO3 solution (500 mL) at 55°C and stirred at 150 rpm for 15 min. Group M_2_T_1_ followed the same procedure using 60 g of NaHCO3.

The relatively high concentrations of sodium bicarbonate (NaHCO3), particularly 30 and 60 g per 50 g of cassava peel, were applied to evaluate the maximum effectiveness of alkaline treatment in reducing HCN levels. These concentrations were used to establish a dose–response relationship and determine the upper limit of detoxification efficiency, providing baseline data for process optimization and future scale‐up.

### 2.4. Determination of HCN Using a UV‐Vis Spectrophotometer

HCN determination was conducted following the method described by Narwati and Setiawan [14]. Total HCN was analyzed using the picrate paper method as described by Ayele et al. [18].

Picrate papers were prepared by immersing 0.3 mm thick filter paper in a 2.5% (*w*/*v*) picrate solution (Sigma‐Aldrich, St. Louis, Missouri, United States) and drying them in a fume hood. The dried papers were cut into 3 × 1 cm strips and affixed to plastic supports measuring 5 × 1 cm with a thickness of 1 mm. Linamarase was obtained through enzyme extraction and purification using gel filtration chromatography (Perkin Elmer 2400 CHN/O Analyzer, United States). For analysis, 0.05 g of sample was mixed with 1 mL of 0.1 M sodium phosphate buffer (Merck, Germany), and 100 *μ*l linamarase in a vial. A picrate paper strip was inserted into the vial, which was immediately sealed and incubated at 30°C for 24 h.

After incubation, the picrate paper was removed and immersed in 5 mL of distilled water for 30 min. A blank was prepared using a picrate paper without sample. Cyanide standard curves were prepared using linamarin (Sigma‐Aldrich, United States) concentrations ranging from 0.2 to 2.4 *μ*M. Absorbance was measured at 510 nm using a UV–Vis spectrophotometer. Phytate content was also analyzed. Dried samples (0.5 g) were extracted with 10 mL of 3.5% hydrochloric acid (HCl) and stirred for 1 h, followed by centrifugation at 3000 × g for 10 min. The supernatant was further centrifuged at 10,000 × g for 10 min. Wade reagent was prepared by dissolving 30 mg iron (III) chloride hexahydrate and 300 mg sulfosalicylic acid in 100 mL of distilled water. Absorbance was measured at 500 nm using a UV–Vis spectrophotometer (Model 1800 PC, Japan).

### 2.5. Data Analysis

The data obtained from the test were then subjected to statistical analysis. The five groups were then subjected to a one‐way analysis of variance (ANOVA) to compare their tear strength. The statistical analysis was conducted with a 95% confidence level and a significance threshold of *p* < 0.05. In order to perform intergroup comparisons between the test and control groups, a post hoc Tukey test was used.

### 2.6. Ethical Approval

The study was approved by the Ethics Review Board of Poltekkes Kemenkes Surabaya (Approval Number No. EA/2317/KEPK‐Poltekkes_Sby/V/2024).

## 3. Results

### 3.1. Average HCN Levels in Cassava Peel Samples

Table 1 below shows that the average levels of HCN in cassava peel samples differed significantly among treatments (*p* < 0.05). The KM_0_T_0_R group exhibited the highest HCN concentration (1.60 ± 0.05 mg/100 g), indicating minimal cyanide reduction during processing. This group did not undergo NaHCO3 treatment prior to heating. The M_1_T_0_R and M_2_T_0_R groups showed moderate decreases in HCN levels, with concentrations of 1.39 ± 0.04 and 1.23 ± 0.07 mg/100 g, respectively. In contrast, substantial reductions in HCN levels were observed in the M_1_T_1_R (0.48 ± 0.03 mg/100 g) and M_2_T_1_R (0.19 ± 0.01 mg/100 g) groups, suggesting that combined treatments involving heating and extended soaking time were most effective for cassava peel detoxification. The progressive decline in HCN levels from KM_0_T_0_R to M_2_T_1_R demonstrates that multistep processing significantly enhances the removal of cyanogenic compounds.

**Table 1:** Mean ± SD of HCN concentration in cassava peel samples.

Sample code	HCN concentration (mg/100 g) *m* *e* *a* *n* ± *S* *D*
KM_0_T_0_R	1.60 ± 0.05^a^
M_1_T_0_R	1.39 ± 0.04^b^
M_2_T_0_R	1.23 ± 0.07^c^
M_1_T_1_R	0.48 ± 0.03^d^
M_2_T_1_R	0.19 ± 0.01^e^

*Note:* Different superscript letters (a–e) indicate significant differences (*p* < 0.05).

Table 1 shows a statistically significant variation in HCN concentrations among all treatment groups (*p* < 0.05). The KM_0_T_0_R group exhibited the highest HCN concentration (1.60 ± 0.05 mg/100 g), indicating that HCN reduction was minimal when no NaHCO_3_ treatment and no extended processing were applied. This result suggests that basic processing alone is insufficient to effectively remove cyanogenic compounds from cassava peel.

Samples subjected to limited processing, namely M_1_T_0_R and M_2_T_0_R, demonstrated moderate but significant reductions in HCN levels, with concentrations of 1.39 ± 0.04 and 1.23 ± 0.07 mg/100 g, respectively. These findings indicate that partial processing contributes to cyanide reduction; however, the extent of detoxification remains limited without additional treatment steps. In contrast, a pronounced decrease in HCN concentration was observed in samples treated with combined processing methods. The M_1_T_1_R and M_2_T_1_R groups recorded substantially lower HCN levels, at 0.48 ± 0.03 and 0.19 ± 0.01 mg/100 g, respectively. The use of distinct superscript letters confirms that these reductions were statistically significant compared with the other treatment groups. The progressive decline in HCN levels from KM_0_T_0_R to M_2_T_1_R demonstrates that multistep processing, involving chemical treatment and extended soaking time, plays a critical role in enhancing the removal of cyanogenic compounds from cassava peel.

### 3.2. Analysis of Differences in Average HCN Levels in Cassava Peel

Data from the HCN examination of cassava tuber skin samples were tested for normality and homogeneity. Fulfillment of both tests is followed by the ANOVA statistical test. One‐way ANOVA test was conducted to determine the difference in HCN levels based on the mass of NaHCO_3_ before and after heating at a stirring speed of 150 rpm for 15 min. ANOVA test of HCN is shown in Table 2.

**Table 2:** Analysis of variance (ANOVA) for the effects of mass variation and heating on HCN concentration in cassava peel.

Source	Type III sum of squares	df	Mean square	*F*	*p* value
Corrected model	7.407^a^	4	1.852	819.907	0.001
Intercept	23.869	1	23.869	10568.651	0.001
Massa and heating	7.407	4	1.852	819.907	0.001
Error	0.045	20	0.002		
Total	31.321	25			
Corrected total	7.452	24			

^a^ *R* ^2^ = 0.994 (adjusted *R* ^2^ = 0.993).

Table 2 shows the ANOVA results indicate that mass variation and heating treatment had a significant effect on HCN levels in cassava peel (*p* < 0.001), with the model explaining 99.4% of the total variance (*R* ^2^ = 0.994).

Table 3 below shows the results of ANOVA of HCN levels.

**Table 3:** Post hoc Tukey HSD analysis of HCN reduction in cassava peel subjected to NaHCO3 treatment and heating.

(I) Sample	(J) Sample	Mean difference (I–J)	Std. error	*p* value
KM0T0	M_I_T_0_	0.2162^a^	0.03006	0.001
	M_1_T_1_	1.1230^a^	0.03006	0.001
	M_2_T_0_	0.3774^a^	0.03006	0.001
	M_2_T_1_	1.4088^a^	0.03006	0.001

^a^Shows a significant difference in the average.

Table 3 shows the Tukey HSD test confirms significant differences in HCN levels between the control group (KM_0_T_0_) and all treated groups (*p* < 0.001), indicating that NaHCO_3_ mass variation combined with heating significantly influences HCN reduction. The highest difference in HCN levels with a mass of NaHCO_3_ of 60 g (M_2_T_1_) with a mean difference to the control group of 1.4088 ^∗^, the percentage reduction in HCN levels by 87.9%, namely to 0.1934 mg/100 g.

## 4. Discussion

The HCN levels of cassava peel treated with stirring in this study were 1.6024 mg/100 g. The high HCN levels of cassava peel in this study are thought to be due to the lack of soaking time in the stirring process and without changing the soaking water. Ukonu et al. explained that soaking in liquid media allows greater extraction of HCN to dissolve in the soaking water. A 4‐h soaking process can remove 20% of the cyanide. In this study, the decrease in HCN has not provided a safe limit for cassava leaves to be consumed. The time used only ranges from 15 to 45 min, so the HCN reduction is still categorized as high, namely > 40 ppm. Ukonu et al. further explained that the decrease in cyanide is more significant if there is a regular change of soaking water within 3–5 days [19].

The range of HCN of cassava peel in this study has also been reported by Amelework and Bairu that the normal range of HCN in cassava ranges from 1 to 130 mg per 100 g dry weight. The HCN of the roots is lower than the leaves and stems of cassava. Cassava roots contain HCN of 1–50 mg per 100 mg of dry matter [20]. The HCN levels in this bitter cassava variety exceed the intake recommended by FAO and WHO, which is 1 mg/100 g dry weight, making its consumption toxic to humans. According to Siritunga and Sayre, the root parenchyma contains 1 to 50 mg/100 g of HCN [21]. Cassava peel soaked in water and stirred in this study caused a hydrolysis reaction. It has been reported that cells that are hypotonic cause water as a soaking solution to cause cells to expand so that hydrolysis reactions occur [22]. The soft structure of cassava peels causes water to enter the cells more easily and makes cyanide easily come out and dissolve in water.

The lowest HCN levels were in the group given 60 g NaHCO_3_ after heating at 55°C (M_2_T_1_). HCN levels with the addition of NaHCO_3_ in this study still left 1.2248 mg/100 g and a decrease in HCN of only 23.56% compared with the levels of cassava peel that were intervened only with stirring for 15 min. Stirring and the addition of NaHCO_3_ contribute to reducing HCN in cassava peels. Research by Narwati and Setiawan showed that the addition of NaHCO_3_ through the stirring method can reduce HCN [14]. Stirring can potentially enhance the process of cyanogenic glucoside hydrolysis by the linamarase enzyme [23]. Kuliahsari et al. explained that cyanide is a toxic substance found in several tubers such as cassava (*Manihot esculenta*), wild yam (*Dioscorea hispida* Dennts), some cereals, and beans. In plants, it can be in the form of cyanogenic glycosides, acetone cyanohydrin, and HCN. Cyanogenic glycosides such as linamarin and lotaustralin belong to secondary metabolic products. The characteristics of cyanogenic glycosides are intermediate polar, water‐soluble, and often accumulate in the vacuoles of plant cells. Cyanogenic acetone and HCN in plant tissues. HCN is a volatile and water‐soluble compound [24]. This indicates that the decrease in HCN levels in cassava peel through stirring for 15 min in the study is thought to be due to the nature of HCN compounds that are easily soluble in water. Joniur et al. explained that cyanogenic glycosides when enzymatically hydrolyzed release HCN [25]. Nyirenda explained that hydrolysis of cyanogenic glycosides is carried out by *β*‐glucosidase to produce the corresponding cyanohydrin, further decomposing and releasing HCN and aldehydes or ketones. It is further explained that the cyanogenic glycosides linamarin (*α*‐hydroxybutyronitril‐*β*‐d‐glucopyranoside) and lotaustralin (ethyl linamarin) are distributed in the vacuoles of cassava cells, whereas the enzyme linamarase is found in the cell wall [26].

The addition of NaHCO_3_ is a modified method in reducing HCN. Increasing the mass of NaHCO_3_ has an impact on the higher HCN reduction. The reduction of HCN with the addition of NaHCO_3_ was also proven in Sari and Nurfajriah′s research. The reduction of HCN with the intervention of NaHCO_3_ solution immersion for 1 h, obtained a decrease in HCN by 22.05% with HCN levels of 41.2656 ppm. The addition of NaHCO_3_ causes the softening of the cassava peel tissue, making it easier for the linamarin contained in the cassava skin to decompose. The process of decomposing linamarin is due to the hydrolysis reaction of the linamarase enzyme. The soaking process through stirring for 15 min in NaHCO_3_ solution at room temperature can break down the plant cell wall [27]. Nasyanka et al. further explained that the soak time needed to get better results is 6–24 h [28].

Difference in HCN level of cassava peel against control group (KM_0_T_0_) shows the difference in HCN levels of cassava tuber skin in each treatment of NaHCO_3_ addition before and after heating against the HCN of cassava tuber skin that was not added with NaHCO_3_, at 150 rpm stirring for 15 min. The highest difference in HCN levels against the control group was found in the group that was given the addition of NaHCO_3_ after 55°C heating, which was 1.409 mg/100 g. This shows that with the treatment of NaHCO_3_ addition with 55°C heating, the HCN of cassava tuber skin decreased by 87.9%. This 87.9% decrease indicates that the heating factor can increase the ability to reduce HCN in addition to the addition of NaHCO_3_. Previous research conducted by Narwati and Setiawan showed that the decrease in HCN in cassava leaves carried out only by adding NaHCO_3_ through stirring was 62.1%. The difference in the increase in HCN reduction with heating intervention was 25.8%. Preprocessing of cassava peel in this study is through the process of slicing small pieces of 2 mm wide cassava peel to expand the surface of cassava peel. The activity continued with washing and soaking in NaHCO_3_ solution. Ayele et al.′s research on cassava preprocessing is not only intended to reduce HCN but also to minimize the loss of cassava leaf nutrients. The HCN reduction obtained with the chopping technique that was mixed at 25°C was 57% [18]. Andama and Oloya explained in their research that a combined method of cassava preprocessing is needed to detoxify HCN before consumption [29]. The effect of soaking time in NaHCO_3_ solution on HCN reduction has been carried out by Triana and Kamila. The results showed that HCN levels can decrease by 84.22% after soaking for 12 h with 20% NaHCO_3_ mass.

The increase in HCN reduction through modification of physical methods, namely heating and stirring of cassava peel in NaHCO_3_ solution, is thought to be the breaking of linamarin bonds due to increased temperature. Mosayyebi et al. explained that linamarin is a cyanohydrin acetone glycoside and its *β*‐bond can be broken by high temperature, high pressure, and enzymatic activity [30]. Linamarinase, which is found in all parts of cassava plant tissue, can decompose linamarin into acetone cyanohydrin and glucose, and then acetone cyanohydrin turns into more toxic cyanide [5].

The HCN produced by cassava results from the hydrolysis of linamarin by the enzyme linamarase, forming an acetone cyanohydrin, which is then converted to release HCN either spontaneously or enzymatically [31]. HCN is a toxic compound that results from the hydrolysis of cyanogenic glycosides [32]. Cyanogenic glycosides are usually O‐*β*‐glycosidic derivatives of *α*‐hydroxynitrile derived from five hydrophobic protein amino acids namely tyrosine, phenylalanine, valine, leucine, and isoleucine [33, 34]. The total HCN levels in cassava peels ranges from 15 to 36 mg of HCN per 100 g fresh weight. Cassava produces two types of glucocyanide compounds, namely linamarin (2‐*β*‐D‐ glucopyranosiloxyl) and lotaustralin (methylbutyronitrile) which are mostly found in the cassava skin. Linamarin accounts for more than 80% of cassava cyanogenic glucosides. It is the *β*‐glucoside of acetone cyanohydrin and ethyl‐methyl‐ketone‐cyanohydrin. When the cyanogenic glucoside undergoes enzymatic hydrolysis, HCN is liberated [7].

The continuous consumption of cassava and its by‐products if improperly processed (not properly cooked, dried or fermented) will cause a series of problems for human health, leading to poisoning and diarrhea that shows clinical symptoms such as mental disorders, muscle paralysis, and depression, respiratory distress, headache, dizziness, blood disorders, thyroid enlargement, brain, central nervous system, heart and kidney damage, nausea, vomiting, diarrhea, abdominal pain, acidosis, heart failure, pulmonary edema, breathing difficulties, bradycardia, hyperlactatemia, hypotension, apnoea, convulsions, coma, opistotonus, jaw stiffness, dilated pupils, or even death [35]. Nyamekye explained that cyanide affects people who often consume cassava over a long period. HCN is easily absorbed in the body. The presence of cyanide in the body affects the body′s cells in using oxygen. HCN will bind to the main iron‐containing enzyme and stop cellular respiration. Exposure to low doses of cyanide can cause heart failure [10].

Nonlethal doses may cause headache, hyperventilation, vomiting, weakness, abdominal cramps, and partial failure of the circulatory and respiratory systems [33]. As reiterated by Harenčár et al., daily consumption of 0.002 mg cyanide per 100 g can cause acute symptoms and chronic symptoms of 0.008 mg cyanide per 100 g (Ľubomír [36]). Panter explained that cyanide poisoning is a serious human health problem, especially in the tropics. It is further explained that cyanide inhibits oxygen utilization and increases anaerobic metabolism leading to lactic acid overload metabolic acidosis and ultimately cell death due to energy deprivation [37].

**Figure 1:**
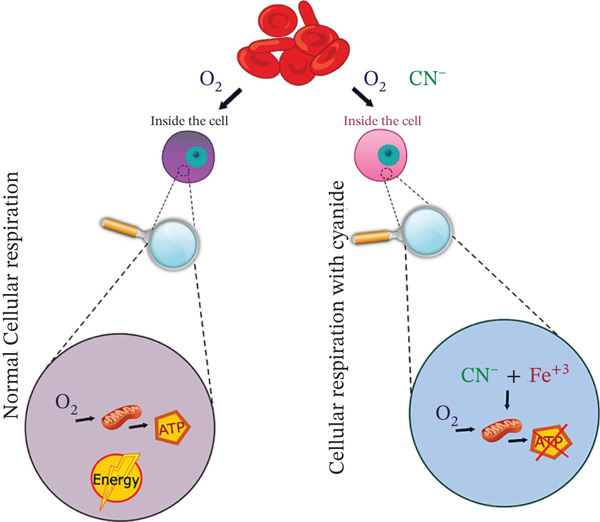
Inhibition of cellular respiration by cyanide in the protein structure of cytochrome C oxidase [36].

HCN in humans is a toxic and fast‐acting substance. Acute (14 days or less), intermediate (15 to 364 days), or chronic (365 days or more) exposure will depend on the amount of dose and duration of time when absorbed orally, through skin, eyes, or by inhalation. HCN has a great affinity for metalloproteins (about 40 enzyme systems) such as cobalt (Co) and iron ions (Fe^3+^) in methemoglobin, as well as cytochrome oxidase and iron ions (Fe^2+^) in hemoglobin. Besides binding to cytochrome c oxidase, cyanide can also contribute to signs of intoxication due to inhibition of catalase, peroxidase, hydroxocobalamin, phosphatase, tyrosinase, ascorbic acid oxidase, xanthine oxidase, and succinate dehydrogenase activities. The inhibitory effect of cellular respiration and the interaction of Fe atoms with CN‐ can be shown in Figure 1.

The HCN of cassava peel in the study was higher than the level recommended by WHO (10 mg HCN/kg body weight). The HCN content of cassava peel indicates that if someone consumes cassava peel as food, it can potentially cause health problems. HCN is the most toxic compound, so it is necessary to limit the consumption of cassava peel other than cassava leaves.

## 5. Conclusion

Heating the NaHCO_3_ solution contributes to the reduction of HCN levels in cassava peel. Although the present study demonstrated that HCN reduction can be achieved through a combination of cutting, stirring, and NaHCO_3_ treatment, further studies are required to determine the optimal HCN reduction by varying soaking time, NaHCO_3_ concentration, and processing modifications.

## Author Contributions

The first author prepared the proposal, researched, analyzed, and wrote the draft manuscript. The second author set the objectives and the experimental design, interpreted the results, and finalized the manuscript. The third author is responsible for the management and coordination of the planning and execution of research activities.

## Funding

This study was supported by Politeknik Kesehatan Kementerian Kesehatan Surabaya, Surabaya, Jawa Timur, Indonesia.

## Ethics Statement

This research was conducted according to the standards and procedures of the Ministry of Health Polytechnic Research Ethics Guidelines.

## Consent

All individuals involved in this study regarding the purpose of the survey fully consented to participate. No animal/human biological elements are included in this study; only plant elements are included.

## Conflicts of Interest

The authors declare no conflicts of interest.

## Data Availability

The data used is primary data, the results of laboratory tests on research samples.
